# Detection of Light Images by Simple Tissues as Visualized by Photosensitized Magnetic Resonance Imaging

**DOI:** 10.1371/journal.pone.0001191

**Published:** 2007-11-21

**Authors:** Catherine Tempel-Brami, Iddo Pinkas, Avigdor Scherz, Yoram Salomon

**Affiliations:** 1 Department of Biological Regulation, The Weizmann Institute of Science, Rehovot Israel; 2 Department of Plant Sciences, The Weizmann Institute of Science, Rehovot, Israel; University of Arizona, United States of America

## Abstract

In this study, we show how light can be absorbed by the body of a living rat due to an injected pigment circulating in the blood stream. This process is then physiologically translated in the tissue into a chemical signature that can be perceived as an image by magnetic resonance imaging (MRI). We previously reported that illumination of an injected photosynthetic bacteriochlorophyll-derived pigment leads to a generation of reactive oxygen species, upon oxygen consumption in the blood stream. Consequently, paramagnetic deoxyhemoglobin accumulating in the illuminated area induces changes in image contrast, detectable by a Blood Oxygen Level Dependent (BOLD)-MRI protocol, termed photosensitized (ps)MRI. Here, we show that laser beam pulses synchronously trigger BOLD-contrast transients in the tissue, allowing representation of the luminous spatiotemporal profile, as a contrast map, on the MR monitor. Regions with enhanced BOLD-contrast (7-61 fold) were deduced as illuminated, and were found to overlap with the anatomical location of the incident light. Thus, we conclude that luminous information can be captured and translated by typical oxygen exchange processes in the blood of ordinary tissues, and made visible by psMRI (Fig. 1). This process represents a new channel for communicating environmental light into the body in certain analogy to light absorption by visual pigments in the retina where image perception takes place in the central nervous system. Potential applications of this finding may include: non-invasive intra-operative light guidance and follow-up of photodynamic interventions, determination of light diffusion in opaque tissues for optical imaging and possible assistance to the blind.

**Figure 1:**
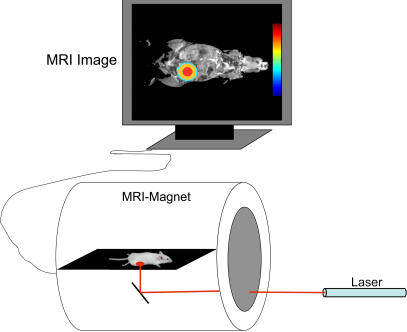
Schematic view of the experimental set up for in-vivo translation of light into an MR-image. The laser pulses are projected onto the pigment-treated animal placed inside the MRI magnet. The illuminated display is physiologically translated in the tissue into a chemical signature and reproduced as an image on the MRI monitor.

## Introduction

Magnetic Resonance Imaging (MRI) is an established, powerful, noninvasive method for tomography of otherwise opaque tissues [1], [2]. While optical imaging techniques are limited by tissue depth, due to absorption, light scattering and photon diffusion processes [3], the study of light diffusion in opaque tissues may now be facilitated based-on the detection of luminosity by Blood Oxygen Level Dependent (BOLD)-MRI, as shown below.

BOLD-MRI provides monitoring of acute changes in tissue oxygenation status, endowing spatiotemporal information [4], as routinely performed in functional fMRI during neuroimaging ([5], [6] and references therein). This technique, based on the magnetic properties of endogenous deoxyhemoglobin (deoxyHb), enables rapid readouts of the dynamic balance between circulating paramagnetic deoxyHb and diamagnetic oxyHb. Image contrast of BOLD-MRI may also be affected by blood flow, carbon dioxide tension, hematocrit, pH or the presence of biphosphoglycerate [7]. Applications of BOLD-MRI include evaluation of tissue oxygen levels [8], studies of angiogenesis [9] and evaluation of pathological ischemia in the kidney [10], brain [11] and heart [12]. In other applications, like neuroimaging [5], [6], hypercapnia/hyperoxia studies [13], and Vascular Targeted Photodynamic Therapy (VTP) [14], BOLD MRI-contrast is artificially manipulated. In VTP, an externally triggered localized photochemical process generates reactive oxygen species (ROS) in the circulation, inducing focal deoxyHb accumulation and BOLD contrast [14] upon oxygen [15] consumption.

Vascular Targeted Photodynamic Therapy is a local treatment modality used to ablate solid tumors upon focal illumination of tumor-bearing animals pretreated with a photosensitizer drug. This treatment involves intravenous injection/infusion of Pd-bacteriochlorophyll derivatives [16] (e.g. WST09 [14], [17]–[21], or WST11 [22], [23]) as photosensitizing drugs, followed by immediate local transdermal or interstitial illumination provided by optic-fiber at matched near-infrared wavelengths (763 or 755nm respectively). Consequently, cytotoxic ROS (mainly superoxide and hydroxyl radicals [24]) are generated in the tumor vasculature, resulting in acute vascular occlusion and blood stasis, development of necrosis and tumor eradication. This therapeutic modality is presently in phase II clinical trials for prostate cancer therapy, in collaboration with Steba-Biotech and Negma France [18].

This study describes how an illuminated tissue and the photochemistry involved in VTP can act together as a detector-screen to translate a spatiotemporal light profile into paramagnetic information imaged by psMRI (Fig. 1). This detector-screen consists of the photo-excited pigment that circulates in the blood vessel network, driving an oxygen-exchange cascade, which culminates with image mapping of light dependent deoxyhemoglobin distribution. The performance of the detector-screen is influenced by its matrix density and uniformity. Biological implications are discussed, and possible practical applications of this new imaging concept are suggested.

## Results

**Figure 2:**
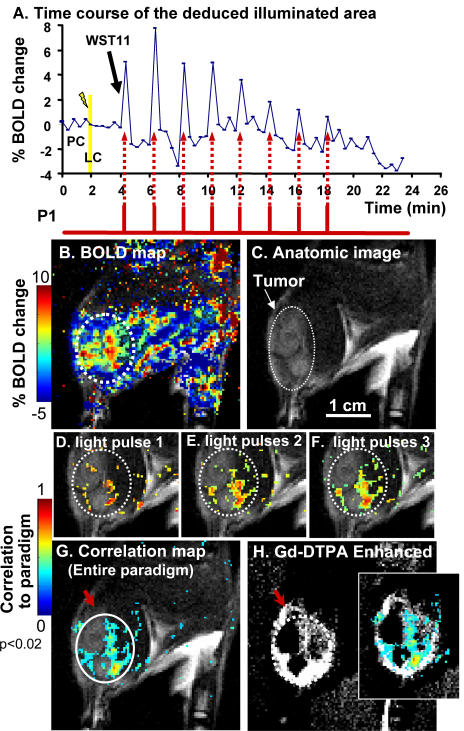
Temporal correlation between illumination and BOLD-contrast changes. A. Time-course of the BOLD response in the deduced illuminated area (white circle in G). The first 4 min included the pretreatment (PC) and the light control (LC) pulses (200 mW/cm^2^). Each time point corresponds to a 12 s T_2_* BOLD-sensitive image. Black arrow indicates WST11 injection to begin photosensitization, using an alternating light∶dark sequence (12 s∶110 s), paradigm P1 (red bars). B. Percent BOLD activation map at t = 6.4 min, overlaid on the anatomic image, C. C. MRI coronal view of the rat thigh, s.c. grafted with MADB106 tumor. A light beam (φ 1 cm) was projected onto the tumor area. The white dotted circle depicts the tumor. D–F. Correlation coefficient maps (p<0.02) obtained after the 1^st^, 2^nd^ and 3^rd^ light pulses respectively, overlaid on the anatomic image. G. Correlation coefficient map of the entire paradigm (60 images) overlaid on the anatomy, allowing deduction of the illuminated field on the psMR image (white circle) that spatially overlaps with the incident light. The colored pixel clusters represent high blood vessel densities and/or larger vessels in the illuminated zone; while the dark areas represent low/no vascularity. The contrast enhancement ratio of illuminated relative to the surrounding areas was 49-fold (average 33±23SD, n = 8). H. Gd-DTPA contrast enhanced imaging (20 min post-treatment) marks functional, permeable blood vessels. The inset shows the correlation map overlaid on the GdDTPA enhanced image. Note: non-illuminated vasculature is not visible on the correlation map (compare red arrows in G versus H). Dashed white circles represent copies of the white circle in G. BOLD-contrast scale bar relates to (B) and correlation scale bar to (D–G).

We hypothesized that if a pigment-treated animal is illuminated, the luminous incident-image displayed on the animal tissue can be translated in vivo into a deoxyHb-dependent BOLD-MRI contrast map, reproducing its spatiotemporal profile. A laser beam (755 nm) was directed at a subcutaneous (s.c.) tumor, grafted to the thigh of a rat, which was placed in the MRI magnet (Fig. 1, Fig. 2). Following the pretreatment (PC) and light (LC) control scans, the animal was i.v. injected with WST11 and the tumor was illuminated by an alternate light∶dark (12 s∶110 s) sequence paradigm (P1). Decreases in MR signal intensity, due to local deoxyHb accumulation, were used to create maps of the BOLD-contrast changes, relative to the PC baseline (see methods). Well-defined BOLD-contrast activation spikes (7–8% higher than PC) demonstrating synchrony with the illumination paradigm were instantaneously observed, while BOLD-contrast reverted to the baseline values during the intervening dark phases, where DeoxyHb was apparently washed out upon re-oxygenation (Fig. 2A). No contrast changes were observed in the PC and LC.

Processing of the collected data into a BOLD-MRI contrast map (for example at 6 min, 2nd pulse, Fig. 2B), overlaid onto the anatomic image (Fig. 2C), appeared to represent the boundaries of the incident display rather poorly, most likely due to low signal to noise ratio, motion or detector-screen artifacts. To overcome this problem, all 60 sequential BOLD activation maps collected were processed into a single map, termed the correlation map. This map was obtained by computing the temporal correlation between BOLD activation and the illumination sequence paradigm (p<0.02), on a pixel-by-pixel basis (see Methods). Pixels whose BOLD contrast responded in synchrony with the illumination paradigm were identified, as illuminated. Repetition of the 12 s light pulses progressively contributed to the image quality and to the reduction of the surrounding noise, as shown for the first three light pulses (Fig. 2D–F). The final correlation map presents pixel clusters which allow deduction of the illuminated zone that overlapped with the anatomic location of the incident light, as determined with the aiming laser beam (Fig. 2G, white circle). Relative to the surrounding area, this zone demonstrated a significantly positive contrast enhancement ratio of 49-fold in this experiment (average 33±23SD, n = 8), whereby false-positive pixels were barely detectable (0.6%, average 1%±0.4SD). The non-uniform distribution of the pixel clusters within the illuminated region may reflect variations in tumor vascular density, as independently verified by GdDTPA-contrast-enhanced MRI of the tumor's vascular functionality. The enhanced pixels of the correlation map, which lie within the illuminated display (white circle Fig. 2H & insert), indeed coincide with the tumor vascular pattern, as confirmed by GdDTPA contrast-enhanced MRI. However, GdDTPA-enhanced areas outside the deduced illuminated zone (red arrow) were not identified in the correlation map (compare red arrows in Figs. 2 G&H). Thus, psMRI features the unique ability to detect and differentiate illuminated, from non-illuminated vascular regions. Additionally, the synchrony between the BOLD response pattern and the pulsed light paradigm illustrates the reversible nature of the photosensitization process (Fig. 2A). Although GdDTPA-enhanced imaging demonstrated vascular functionality at the treatment site following the eight consecutive 12 s light pulses (Fig. 2H), 90% tissue necrosis was histologically observed in the tumor 24 h later (not shown). Yet, negligible necrosis was observed following a single 12 s light pulse of 200 mW/cm^2^ (2.4 J/cm^2^), equivalent to 0.6–4% of the light dose routinely used in standard therapeutic VTP protocols (60–360 J/cm2) used to induce acute, irreversible blood stasis before the end of the treatment [25], [26]. The gradual decline in spike height seems to reflect WST11 clearance and possibly development of vascular photo-damage (Fig. 2H). No significant correlation was found when the BOLD contrast results were tested against delayed illumination paradigms, eliminating the possibility of a random or deferred relationship.

**Figure 3:**
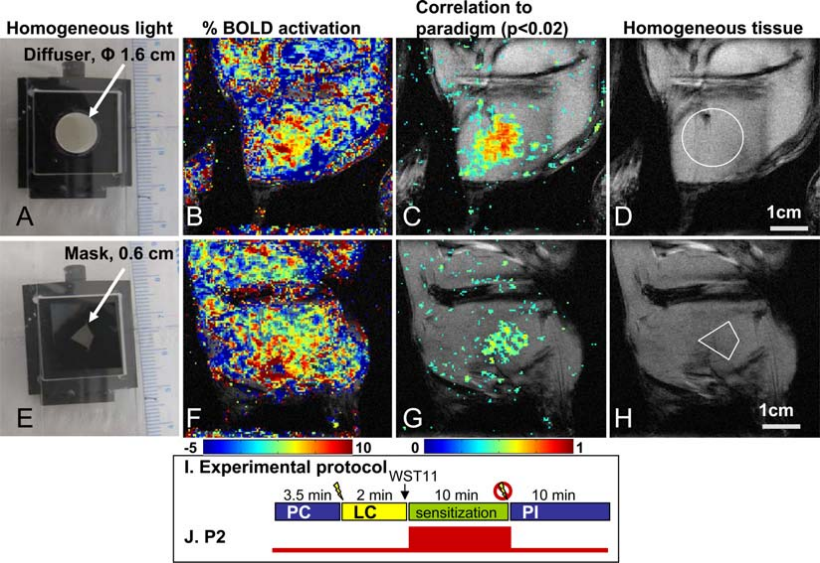
Shape-recognition of illumination field by BOLD-contrast changes: P2 paradigm. Homogeneous light (55 mW/cm^2^) was delivered via a diffuser onto the rat thigh. With the use of respective masks the light beam-cross section was circular (φ 1.6 cm, A–D) or kite-shaped (0.6 cm length, E–H). B&F. Representative BOLD-contrast activation maps acquired at the end of the photosensitization phase are overlaid on the anatomic image and D&G. are the respective correlation coefficient maps (p<0.02). Colored pixel clusters on the anatomic image outline the deduced shape of the light field. Contrast enhancements of the deduced circle and the kite shapes were respectively 21 and 24-fold higher than their neighboring surroundings. D&H. Locations and shapes of the projected light fields on the respective anatomic images are deduced from the above correlations (white shapes). I. The BOLD-MRI protocol, using an acquisition time of 25 s/image yielded a total of 45 (A–D) or 40 images (E–H). J. Paradigm P2 consists of a single 10 min illumination. PI = Post-illumination.

The non-uniform vascular pattern of the tumor was found to dominate the BOLD activation map, creating a detector-screen artifact that interfered with the recognition of the spatiotemporal profile of the incident light beam. To improve the performance of the tissue as a detector screen we chose a different model, where a light beam was directed onto healthy, striated muscle tissue in the rat thigh. This tissue contains a rich, uniform vascular matrix. Light beams with a circular, or kite-shaped cross-section were created by placing respective masks in the light path (Figs. 3 A&E). In these experiments, we used a 10 min illumination paradigm, P2 (55 mW/cm^2^, 33 J/cm^2^, Fig. 2J). As in the tumor model, the anatomic location of these light fields were fully ascertainable by the correlation maps (Figs. 3 C&G), calculated from the BOLD-contrast maps (Figs. 3 B&F). The contrast enhancement ratio of the deduced illuminated areas was 23±2SD (n = 3) fold higher than in the respective non-illuminated surroundings. These results suggest that when detector uniformity increases, as in the case of the capillary bed of striated muscle tissue, the accuracy of the psMRI image increases. While image location coincided with the incident light, deviations from the circular shape of the display were observed on the psMR image (Fig. 3C). This was probably related to a downstream effect of deoxyHb. The magnified size of the registered images, relative to the original, may stem from inherent photon diffusion and tissue light scattering. A slight degree of photo-damage, marked by edema and scattered myonecrosis, was histologically observed in the muscle 24 h post illumination. However, it should be noted that the vasculature of normal healthy tissues is generally less sensitive to VTP, as compared to tumor tissue [19].

## Discussion

This study provides evidence that a luminous display, projected onto tissues of animals treated with a photosensitizer, can be captured, viewed and recognized by psMRI. Furthermore, it is possible to distinguish illuminated from non-illuminated blood vessels (Fig. 2), due to endogenous deoxyHb and its reactivity in the illuminated regions. Under the conditions used, an increase in signal to noise ratio of the correlation maps was gained with increased light pulse number, improving the clarity of the psMR image. In contrast, a decline in the correlation values was observed at the same time (Fig. 2 D–G), possibly due to accumulating biological effects (sensitizer clearance and/or photo-damage); this will have to be considered when programming future stimulation paradigms. It is anticipated that by manipulation local phototoxicity, forthcoming improvement of experimental conditions, optimization of MRI and better image processing algorithms can reduce the light pulse energy requirements for its imaging. These improvements will provide an increase in the efficiency of BOLD contrast recording and, subsequently, minimize, or fully eliminate imaging-associated vascular damage. In the case of the therapeutic VTP protocol, whereby target tissue destruction is the objective, damage contributed by simultaneous psMR imaging becomes irrelevant. These predictions are compatible with previous findings in our lab, which demonstrated reversible enzymatic and cellular processes induced by low levels of photosensitized ROS at nondestructive, physiological conditions [27].

When comparing the basis of a BOLD contrast response in psMRI and fMRI, a few points must be considered. Although the response to the light signal is instantaneous in psMRI, the response to neuronal stimulation by fMRI is delayed [28], [29]. This stems from the fact that in psMRI, deoxyHb accumulation is coupled to oxygen consumption by photon-capture of the sensitizer and diffusion-limited dissociation of oxyHb. In contrast, in fMRI-based neuroimaging, deoxyHb accumulation is coupled to stimulation by neuronal input via metabolic consumption of oxygen with a slower hemodynamic response that develops in a matter of seconds.

In summary, the displayed incident image is captured by the tissue as an invisible chemical signature, represented by the spatiotemporal distribution of photogenerated deoxyHb. The dynamic presentation by psMRI of a BOLD contrast map permits recognition of the original incident display, in analogy to visual perception by the brain. The quality of the psMR image is affected by the density and uniformity of the vascular detector-screen (Fig. 3). Furthermore, it is suggested that living organisms have the capacity to detect such light imprinted images, by a circulating detector-pigment (administered or ingested) in tandem with de-saturation of ubiquitous Hb, in a reaction that is unrelated to the visual process. The question of whether endogenous pigments in animals may reflect on environmental luminosity, in a similar manner, as a mode of light detection, remains open. Preliminary results show promising results in the acquisition and imaging of three-dimensional optical information from the vascular detector screen by psMRI.

We envisage psMRI as a means of tracking BOLD contrast changes, following sub-lethal photosensitization in normal tissues, where reduced photo-damage is expected. Immediate applications of this phenomenon may include intra-operative light guidance and a follow-up of photodynamic interventions, as well as a unique option for investigating light diffusion during the development of optical imaging in opaque tissues. Other possibilities may relate to the study of hemodynamic response to light-generated oxygen sinks and, possibly, also assistance to the blind.

## Methods

### Tumor model

MADB106 rat mammary carcinoma cells were cultured in RPMI medium, supplemented with 10% heat inactivated fetal calf serum, 1% non essential amino acids, 100 U/ml penicillin, 0.1 mg/ml streptomycin, 0.25 µg/ml amphotericin B, 2 mM L-glutamine and 1 mM pyruvate (Biological Industries, Kibbutz Beit Haemek, Israel), in an atmosphere of 8% CO_2_ at 37°C. Cells (2×10^6^ cells in 60 µl saline) were grafted s.c. to the thigh of female Fisher rats and the tumors were allowed to grow to treatment size of ∼1 cm. All protocols were approved by the Institutional Animal Care and Use Committee.

### Normal tissue model

The thigh quadriceps muscle of Wistar female rats was used as a normal tissue model.

### Anesthesia and experimental setup

Rats were anesthetized (i.p Ketamine 100 mg/kg, Diazepam 7.5 mg/kg), shaved and positioned supine/laterally above the tumor or thigh, and the examined leg was fixed to the plexiglass tray with adhesive tape to minimize motion artifacts.

### Light source

Illumination was provided by a 1W, 755 nm diode laser, equipped with a 3 mW aiming laser beam (660 nm) (CeramOptec, Germany). Light was delivered with a cleaved bare optic fiber projecting a light field of (φ 1 cm) with Gaussian light distribution onto the skin of the rat (Fig. 2). Alternatively, the light beam was delivered through a diffuser (HoloOr, Rehovot, Israel), creating a circular (top hat cross section) homogeneous light field (φ 1.6 cm) on the animal's skin (Fig. 3). Light delivery to the treated rat inside the MRI magnet was remotely controlled by an electronic inline shutter (Ocean Optics, Dunedin, Fl. USA), gated with the magnetic resonance image scan acquisition.

### Photosensitization protocol

The entire treatment protocol was conducted inside the MRI magnet, where the anesthetized rat was placed in position (Fig. 1). Sequential coronal gradient echo T_2_* weighted images, sensitive to BOLD-contrast (40–60 images/experiment), were acquired during the following four experimental steps: (i) Pretreatment Control (PC); (ii) Light Control (LC) with illumination only. The target tissue was illuminated from below the animal at the indicated fluency rate and duration through a hole in the plexiglass tray holding the rat; (iii) Photosensitization, triggered by bolus injection into the tail vein of 10 mg/kg WST11, dissolved in saline, by remote control through a previously placed catheter, while illumination proceeded according to the preset paradigm indicated in the individual experiments; and (iv) Post-illumination (PI), during which the light is switched off.

Two illumination paradigms, synchronized with image acquisition, were used: P1, consisting of repeated light∶dark periods (12 s∶110 s, a single image/light period); and P2, consisting of continuous 600 s illumination (16 images/light period). The light energies (2.4–33 J/cm^2^) are indicated for the individual experiments.

### MRI set up

BOLD-contrast: Sequential coronal gradient echo T_2_* weighted images, sensitive to BOLD-contrast, were acquired during all four experimental steps by using a horizontal 4.7T Biospec spectrometer (Bruker, Germany) and a 7.5 cm volume coil with the following parameters: Scan acquisition times (12 s or 25 s), as indicated in the individual experiments, inter-scan time interval 9–12 s, TR/TE/α 100 ms/10 ms/30°, in plane resolution 430 µm, matrix 128×128, FOV 5.5 cm. Three slices (1.5 mm thickness) were collected in tumor or muscle tissue. The data presented show the slice positioned closest to, but just below the skin.

### GdDTPA enhanced contrast

Following the VTP protocol, while the rat remained in position, Gd-DTPA (0.1 mmol/kg, Magnevist, Schering, Berlin, Germany) was injected i.v. to examine tumor vascularity, using the same geometry as above with the following parameters: T1 weighted spin echo sequence: TR/TE 140/9 ms, one scan. Due to its small molecular weight, GdDTPA leaks from permeable vessels, enhancing MRI signal from these areas.

### Data Processing

In house programs for data processing were conducted with Matlab 7.1 (The MathWork, Inc., Natik, MA.).

BOLD-contrast change maps: BOLD-contrast maps relative to the average PC control baseline were calculated, pixel-by-pixel, from sequential T_2_* weighted images (one map per time point, total 40–60 per experiment, as indicated in the individual experiments).

BOLD activation (%) = (1–BOLD image/average of BOLD images in PC)×100.

In these experiments the loss in MR signal intensity is presented as a gain in BOLD-contrast. Changes in BOLD-contrast maps are color-coded according to the attached color scale in a range of −5 to +10%.

Correlation coefficient maps were extracted, by normalized, pixel-by-pixel, correlation between the light paradigm and the respective BOLD-contrast maps (calculated over the entire experiment, 40–60 maps). The correlation coefficients are presented in the interval of 0 to 1, where 1 stands for full agreement between data and paradigm. Only correlation coefficients that matched a p-value <0.02 are displayed in the maps. If the significance p(i,j) is small, then the correlation R(i,j) is significant, so as to discard hazardous probability of synchrony. P-values were calculated to test the hypothesis of no correlation. The respective values in the maps were color-coded, according to the attached color scale and overlaid on the anatomical image.

Contrast enhancement was calculated from the correlation coefficient maps (p<0.02) as the ratio between densities of positive pixels in the illuminated area and the rest of the image.

False positive pixels in the non-illuminated area refer to the percentage of pixels that correlated with the paradigm (p<0.02) outside the illuminated area.

### Histology

Target tissues were excised from euthanized rats 24 h after treatment, fixed in 3.8% formaldehyde, followed by standard histological preparation and hematoxylin eosin staining. Pathological evaluation was conducted by Dr. O. Brener, from the Weizmann Institute Pathology Service Unit.
